# Effects of chromium picolinate on glycemic control and kidney of the obese Zucker rat

**DOI:** 10.1186/1743-7075-6-51

**Published:** 2009-12-10

**Authors:** Mahmood S Mozaffari, Rafik AbdelSayed, Jun Yao Liu, Hereward Wimborne, Azza El-Remessy, Ahmed El-Marakby

**Affiliations:** 1 Department of Oral Biology, School of Dentistry, Medical College of Georgia Augusta, Georgia 30912, USA; 2 Department of Oral Health and Diagnostic Sciences, School of Dentistry, Medical College of Georgia, Augusta, Georgia 30912, USA; 3 Clinical and Experimental Therapeutics, College of Pharmacy, University of Georgia, Augusta GA 30912, USA

## Abstract

**Background:**

Chromium picolinate (Cr(pic)3) is advocated as adjuvant therapy for impaired glycemic control, despite concerns for DNA damage. Potential toxicity of Cr(pic)3 should be greater for the kidney that accumulates chromium. Therefore, we tested the hypothesis that Cr(pic)3 treatment of obese Zucker rats (OZR) exacerbates renal abnormalities associated with dysglycemia.

**Methods:**

Male OZR were treated with diets lacking or containing 5 and 10 mg/kg of chromium, as Cr(pic)3, for 20 weeks; lean Zucker rats (LZR) served as controls. Glycemic and renal effects of Cr(pic)3 were determined in the context of indices of oxidative stress and inflammation.

**Results:**

The OZR displayed increased fasting plasma glucose and insulin in association with enlarged pancreatic islets exhibiting collagen and periodic acid Schiff-positive deposits compared to LZR; Cr(pic)3 treatment did not affect these parameters. The OZR, irrespective of Cr(pic)3, excreted more albumin than LZR. Also, other indices of renal function or histopathology were not affected by Cr(pic)3 treatment. Urinary excretion of 8-hydroxydeoxyguanosine (8-OHdG), an index of oxidative DNA damage, was greater in the OZR than LZR; dietary Cr(pic)3 treatment attenuated 8-OHdG excretion. However, immunostaining of kidney for 8-OHdG revealed similar staining pattern and intensity, despite significant renal accumulation of chromium in Cr(pic)3-treated groups. Finally, increased renal nitrotyrosine and cyclooxygenase-2 levels and urinary excretion of monocyte chemoattractant protein-1 of OZR were partially reversed by Cr(pic)3 treatment.

**Conclusion:**

Dietary Cr(pic)3 treatment of OZR does not beneficially influence glycemic status or increase the risk for oxidative DNA damage; rather, the treatment attenuates indices of oxidative stress and inflammation.

## Introduction

The worldwide epidemic of obesity is a major predisposing factor for the ever increasing prevalence and incidence of glucose intolerance and type 2 diabetes [1,2]. In turn, obesity and impaired glucose tolerance/type 2 diabetes markedly increase the risk for development of renal and cardiovascular complications [3,4]. Further, obesity is known as a pro-inflammatory state thereby contributing importantly to eventual target organ manifestations and associated morbidity and mortality [5,6].

Of various animal models of obesity and type 2 diabetes mellitus, the obese Zucker rats (OZR) have been used extensively for studies focused on consequences of the disease and the contributing mechanisms. The OZR have an autosomal recessive mutation of the *fa *gene encoding the leptin receptor. The OZR display marked obesity, dyslipidemia, severe insulin resistance, increased oxidative stress and a proinflammatory state compared to the lean Zucker rats (LZR) [7-9]. Thus, the OZR can serve as a useful animal model for determination of potential influences of therapies aimed at prevention or attenuation of obesity/type 2 diabetes-related abnormalities.

The nutritional supplement, chromium picolinate (Cr(pic)3), contains trivalent chromium which is chelated to three picolinic acid molecules to increase its bioavailability compared to non-chelated forms (e.g., chromium chloride). It is widely used because of claims that it exerts antidiabetic and weight-reduction effects [10-12]. Improvement in glycemic status, in turn, should reduce oxidative stress and the proinflammatory conditions associated with obesity/type 2 diabetes thereby ameliorating target organ complications such as nephropathy. On the other hand, other studies have raised concerns regarding the safety of Cr(pic)3 as they indicate that the formulation increases the risk for DNA damage [13-16]. If indeed the formulation exerts adverse effects in vivo, its potential toxicity would be expected to be more prominent for the kidney which serves not only as its major route of elimination but also accumulates chromium [17-19]. Thus, long-term effects of the formulation need to be established utilizing animal models of the disease for which its use is advocated. Therefore, we tested the hypothesis that, despite improvement in glycemic status, chronic treatment of OZR with Cr(pic)3 causes significant renal accumulation of chromium with adverse consequences for kidney function and structure. Accordingly, renal effects of the chromium formulation were determined in the context of indices of oxidative stress (e.g., tissue nitrotyrosine), inflammation (e.g., urinary excretion of monocyte chemoattractant protein-1 (MCP-1), renal expression of cyclooxygenase-2 and tissue CD68 positive histiocytes) and oxidative DNA damage (e.g., urinary excretion and tissue 8-hydroxydeoxyguanosine (8-OHdG)).

## Methods and materials

Male obese and lean Zucker rats (OZR and LZR, respectively) were obtained form Harlan Laboratories at about 6 weeks of age. The animals were housed in the laboratory animal facilities at the Medical College of Georgia that are controlled for humidity (60% ± 5%), temperature (24° ± 1°C) and light cycle (6 AM to 6 PM). Two days after arrival, the OZR were randomly assigned to either remain on the regular rodent diet (Harlan Teklad diet number 8604) or switched to the 8604-based diet that was supplemented with 5 or 10 mg/kg of chromium as Cr(pic)3 (i.e., OZR; 5 Cr and OZR; 10 Cr, respectively; Harlan Teklad diet numbers 07602 and 07603, respectively; n = 10 animals per group); LZR (n = 10) were provided with the 8604 diet (without supplemental chromium). One untreated OZR and one OZR; 5 Cr died before terminal measurements at about 26 weeks of age. Unless otherwise specified, the animals had free access to food and water throughout the studies. Based on measurement of food intake, the 5 and 10 mg/kg chromium diets provided chromium at doses of 0.19 ± 0.02 and 0.41 ± 0.02 mg/kg/day (or 1.58 ± 0.16 and 3.29 ± 0.12 mg/kg/day of Cr(pic)3, respectively); the chromium intake in this study is similar to other studies utilizing rodents [20-22]. The use of animals for these studies conformed to the institutional guidelines for the care and use of laboratory animals.

At about 26 weeks of age, tail-cuff hemodynamics were measured and two consecutive 24-hour urine samples collected from each animal; urine samples were used for determination of urinary electrolytes, albumin, 8-OHdG and MCP-1 excretions. Further, hemoglobin A1c levels were measured using a drop of blood from the tail (Bayer HealthCare - Diabetes Care, Sunnyvale, California). For determination of plasma glucose and insulin concentrations, the animals were fasted overnight and blood samples were obtained from the tail. Plasma glucose concentration was measured using a Beckman glucose analyzer. On the other hand, plasma insulin concentration was measured using an insulin ^125^I radioimmunoassay Kit (MP Biomedicals, LLC; Solon, OH). The data were used to calculate the insulin resistance Homeostatic Model Assessment (HOMA) index as follows: (FPI × FPG)/22.5 where FPI and FPG denote fasting plasma insulin (μU/ml) and fasting plasma glucose (mmol/l), respectively [23].

After collection of metabolic data and measurement of systemic hemodynamics, the animals were anesthetized with sodium pentobarbital (45-50 mg/kg; i.p.) and renal tissue procured for histopathological (i.e., 10% formalin-fixed), Western and slot blot analyses (frozen in liquid nitrogen) and chromium measurement (air-dried). Also, the pancreas was fixed in buffered formalin for histopathological examination.

Formalin-fixed and paraffin-embedded tissue (i.e., kidney or pancreas) blocks were cut in 5 μm sections followed by staining (e.g., hematoxylin-eosin (H&E), Masson Trichrome or periodic acid Schiff (PAS)); frozen sections were used for Oil-Red-O staining [8]. Immunohistochemical staining was carried out utilizing mouse anti- 8-OHdG primary antibody (Oxis Research, Foster City, CA) and biotinylated goat anti-mouse secondary antibody (Vector Labs, Burlingame CA).

### Western and slot blot analysis

To determine renal cyclooxygenase-2 (COX-2) and nitrotyrosine levels, frozen renal tissue was pulverized and added to the isolation buffer (10 mM triethanolamine, 250 mM sucrose, PH 7.6, 1 μg/ml leupeptin, PMSF (2 mg/ml), sonicated and sodium dodecyl sulfate added to a final concentration of 1% prior to centrifugation; the supernatant was used for protein assay (Biorad protein assay DC kit). Standard protocols were used for Western blot analysis as described previously (i.e., 10% gels, electrophoretic protein transfer to nitrocellulose membrane, rabbit anti-COX-2 polyclonal antibody (Cayman, Ann Arbor, Michigan), secondary antibody (goat anti-rabbit IgG, Cell Signaling, Danvers, MA) and detection by enhanced chemiluminescence) [24,25]. Slot-blot analysis was used to measure the nitrative stress marker: nitrotyrosine. As described previously [26], samples were immobilized onto a nitrocellulose membrane using Slot Blot apparatus (BioRad). After blocking, membranes were reacted with antibodies against nitrotyrosine (Calbiochem), followed by determination of optical density. The COX-2 and nitrotyrosine data were corrected for β-actin and expressed as percent of the LZR group.

### Assays

Urinarysodium and potassium were measured (Easy Electrolytes, Medica Corporations, Bedford, MA) and used to calculate excretion rates. Urine osmolality was measured by an osmometer. Urinary albumin excretion was measured using the Nephrat ELISA kit (Exocell, Philadelphia, PA). Urinary and plasma creatinine were measured (Cayman Chemicals and Biovision, respectively) and used to calculate creatinine clearance rate (an index of the glomerular filtration rate). Urinary excretions of 8-OHdG and MCP-1 were measured using ELISA kits [(Northwest Life Science Specialties, LLC; Vancouver, WA) and (BD Bioscience, San Jose, CA), respectively]. All parameters were determined on two consecutive 24 hour urine samples and average values recorded for each animal. Analysis of kidney chromium content was carried out by the Wisconsin State Laboratory of Hygiene using Inductively Coupled Plasma Mass Spectrometry (ICP-MS) technology.

### Statistics

All data were analyzed by the analysis of variance (ANOVA). Duncan's post hoc test was used for comparison of mean values (significance of criteria of p < 0.05). Data are reported as means ± SEM.

## Results

### Physical features

All rats displayed significant increase in body weight during the course of the study although the OZR, irrespective of Cr(pic)3 treatment, gained significantly more weight than their lean controls (Figure 1A). As an index of skeletal growth, the length of femur and tibia were measured in experimental groups. While obesity was associated with slight reductions in the length of both the tibia and the femur, Cr(pic)3 treatment, per se, did not influence these parameters (Table 1). Kidney weight was greater in the OZR, irrespective of dietary chromium treatment, than LZR; however, kidney to body weight ratio was similar among the groups (Table 1). Further, systolic blood pressure, diastolic blood pressure and mean arterial pressure were similar among the groups (Figure 1B). Also, no differences were noted in heart rates among the groups (average of 462-485 beats/min).

**Table 1:** Physical and metabolic features of experimental groups.

	LZR	OZR	OZR; 5 Cr	OZR; 10 Cr
Femur length (mm)	37.5 ± 0.5*	33.7 ± 0.6	32.8 ± 0.3	32.6 ± 0.4
Tibia length (mm)	42.9 ± 0.3*	40.9 ± 0.2	40.3 ± 0.2	40.8 ± 0.4
Kidney weight (g)	3.44 ± 0.16*	4.74 ± 0.22	4.44 ± 0.2	5.28 ± 0.3
Kidney to body weight (mg/g)	7.04 ± 0.34	6.33 ± 0.21	6.10 ± 0.39	6.92 ± 0.39
				
Plasma glucose (mg/dl)	130.8 ± 5.0*	165.0 ± 8.9	175.0 ± 7.1	176.5 ± 5.5
Plasma insulin (μU/ml)	21.4 ± 1.5*	84.2 ± 4.6	73.3 ± 6.6	80.0 ± 9.9
HOMA index	8.1 ± 1.2*	33.8 ± 2.9	31.8 ± 3.3	34.2 ± 3.9
HgA1c (%)	4.6 ± 0.1	4.9 ± 0.1	4.6 ± 0.2	4.9 ± 0.1
				
Water intake (ml/day)	32.6 ± 0.7	38.7 ± 2.2	44.7 ± 3.8#	47.0 ± 3.1#
Food intake (g/day)	25.4 ± 1.3	29.7 ± 1.4	28.7 ± 2.9	31.2 ± 1.4
Urine excretion (ml/day)	17.6 ± 1.1	23.3 ± 2.1	28.4 ± 3.3#	28.3 ± 3.0#
Urine excretion to fluid intake ratio (%)	54.4 ± 3.0	62.0 ± 3.9	62.9 ± 3.4	59.6 ± 4.2
Urine Osmolality (mOsmol/kg)	1615 ± 72	1655 ± 96	1372 ± 128	1474 ± 102
Sodium excretion (μEq/g food/day)	78.3 ± 5.8	104 ± 10.8#	82.3 ± 4.4	97.1 ± 6.4
Potassium excretion (μEq/g food/day)	231 ± 16	315 ± 57.5	242 ± 13	227 ± 18.6
Creatinine clearance (ml/min)	0.66 ± 0.12	0.60 ± 0.06	0.72 ± 0.09	0.69 ± 0.08
Albumin excretion (mg/day)	41.4 ± 5.4*	70.4 ± 7.2	69.2 ± 7.4	61.6 ± 7.8

* p < 0.05 compared to other groups.

# p < 0.05 compared to the LZR group.

**Figure 1:**
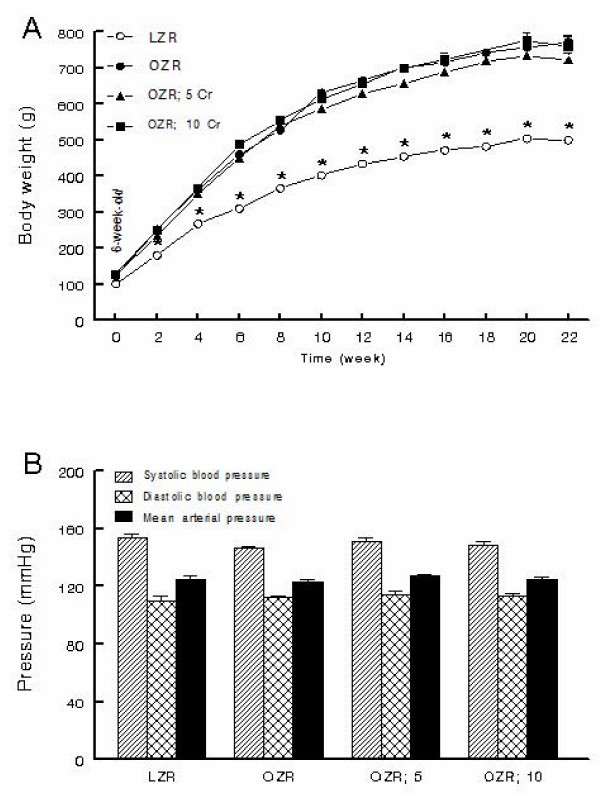
**Body weight and blood pressure**. Panel A shows time-course of changes in body weight while panel B shows blood pressure of lean Zucker rats (LZR), untreated obese Zucker rats (OZR) and OZR treated with diets containing 5 and 10 mg/kg of chromium as Cr(pic)3 (i.e., OZR; 5 Cr and OZR; 10 Cr, respectively). The treatments were initiated at 6 weeks of age (i.e., time 0). Data are means ± SEM of 9-10 animals/group. * p < 0.05 compared to the other groups at the same time point.

### Glycemic status

**Figure 2:**
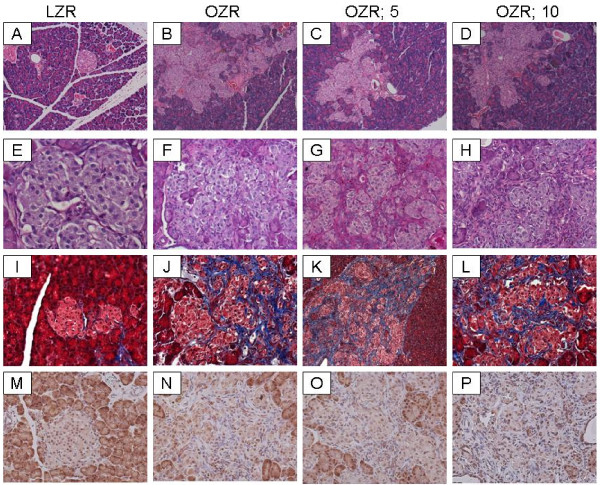
**Pancreas histopathology**. Panels show representative H&E-stained sections (A-D; 100×), PAS-stained sections (E-H; 200×) and trichrome-stained section (I-L; 200×) of pancreas from experimental groups as described in Figure 1. Also shown is the immunohistochemical localization of 8-OHdG in pancreas of experimental groups (Panels M-P, 200×)

The OZR displayed greater fasting plasma glucose (about 30%) and plasma insulin (about 400%) concentrations compared to their lean controls (Table 1). As a result, the HOMA insulin-resistance index was markedly elevated in the OZR, irrespective of Cr(pic)3 treatment, than LZR (Table 1). On the other hand, hemoglobin A1c levels were similar among the four experimental groups (Table 1). Microscopic examination of LZR pancreas revealed well-preserved pancreatic exocrine lobules consisting of packed acinar units which are interspersed by sharply-demarcated, small islets of Langerhans (Figure 2, panel A). Cytologic and nuclear details were bland and uniform in shape, size and staining characteristics. Microscopic examination of pancreas for OZR, OZR; 5 Cr and OZR; 10 Cr groups revealed enlarged, hyperplastic islets of Langerhans (of varying sizes) exhibiting benign cellular proliferation which resulted in irregular and "jagged" peripheral outline (Figure 2, panels B-D). The islet cellular elements were separated by variable aggregates of extracellular deposits; however, the sizes or cytologic and nuclear details of pancreatic islets were generally similar among the three groups of OZR. As shown in Figure 2, while there was minimal staining of the LZR pancreas for PAS (panel E), those of OZR groups (panels F-H), irrespective of Cr(pic)3 treatment showed prominent patchy areas of PAS-positive material. Similarly, trichrome-stained tissue sections from LZR group revealed uniform well-preserved, small collagen-free pancreatic islets (Panel 2, panel I). On the other hand, Trichrome-stained tissue sections from OZR, OZR; 5 Cr and OZR; 10 Cr groups revealed prominent (and generally similar) intercellular deposits of collagen fibers confined within hyperplastic pancreatic islets (Figure 2, panels J-L). These histopathological findings are consistent with the interpretation of islet hyperplasia with interstitial fibrosis.

### Kidney function and structure

The OZR tended to consume more water and excreted more urine than their lean counterparts (Table 1). The Cr(pic)3-treated OZR showed mild increases in water intake and urine excretion. However, the ratio of urine output to water intake was similar among the groups (Table 1). Urine osmolality was similar between the LZR and OZR rats irrespective of Cr(pic)3 treatment (Table 1). The untreated OZR group showed a mild increase in daily excretion of sodium and potassium (normalized to food intake) than LZR; dietary Cr(pic)3 treatment did not affect these parameters (Table 1). Also, creatinine clearance was similar among the groups (Table 1). However, daily albumin excretion was greater in OZR, irrespective of Cr(pic)3 treatment, than LZR (Table 1).

**Figure 3:**
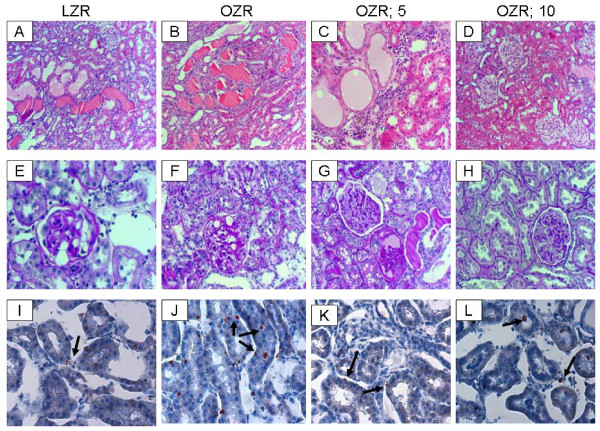
**Kidney histopathology**. Panels A-D show representative H&E-stained while panels E-H show representative PAS-stained kidney sections from experimental groups as described in Figure 1 (100× and 200×, respectively). Also shown are representative panels for Oil-Red-O stained renal tissue from experimental groups (panels I-L, 200×; arrows point to lipid droplets).

Hematoxylin and eosin-stained sections from all groups showed histopathologic changes with varying degrees of intensity (Figure 3, panels A-D). Microscopic examination of tissue sections from LZR and OZR groups revealed histomorphologic changes including tubular dilation, eosinophilic protein casts and minimal interstitial chronic inflammation in 60% vs. 100% of LZR and OZR groups, respectively. Tissue sections from OZR; 5 Cr group showed changes in 75% of subjects with diffuse tubular dilation, tubular protein casts, interstitial chronic inflammation and cystic tubular formations. Tissue sections from the OZR; 10 Cr group showed a mix of histologic grades of intensity with pathologic changes in 80% of subjects ranging from mild (40%) to moderate (20%) and severe (40%; Figure 3D).

For further assessment of renal histopathology, PAS stain was performed to evaluate deposits within the basal lamina surrounding and within the mesangium of the glomerular vascular tuft as well as the interstitial blood vessels (Figure 3E-H). Sections were assigned "mild" when there were focal intraglomerular deposits in 10% or less of glomeruli or interstitial vessels. Sections were assigned "moderate" when there were patchy intraglomerular deposits or interstitial vessels in 20-50% of these structures. Sections were assigned "severe" when there were diffuse intraglomerular deposits and in interstitial vessels involving > 50% of these structures. Sections from LZR showed mild and focal intraglomerular deposits in 20% of subjects. Sections for OZR showed patchy moderate PAS-positive intraglomerular deposits in 80% of subjects. The OZR; 5 Cr group showed diffuse intraglomerular PAS-positive deposits in 70% of subjects while the OZR; 10 Cr group showed mild and patchy intraglomerular PAS-positive deposits in 75% of subjects.

We next performed Masson's trichrome stain on renal tissue to help highlight glomerular or interstitial fibrosis. Tissue sections from LZR and OZR groups did not show trichrome-positive fibrotic changes. However, 60% of kidneys from the OZR; 5 Cr group and 80% of subjects from the OZR;10 group showed few scattered patchy foci of interstitial fibrosis (data not shown).

Oil-Red-O stain was performed to help reveal cellular fat deposits. All groups showed the presence of fat droplets; however, fat droplets were more numerous in the untreated OZR group (Figure 3I-L, arrows).

### Indices of nitrosative/oxidative stress and inflammation

**Figure 4:**
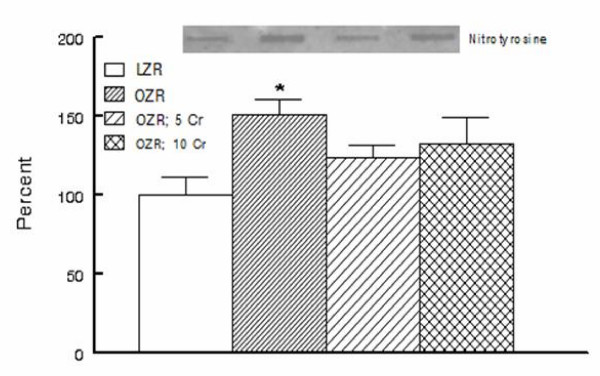
**Kidney nitrotyrosine content**. Bar graphs show renal tissue nitrotyrosine level in experimental groups as described in Figure 1; data are expressed as percent of the LZR group. Also shown are representative blot for each group. Data are means ± SEM of 7-9 animals per group. * p < 0.05 compared to the LZR group.

Tissue nitrotyrosine is considered as a stable footprint of oxidative stress. As shown in Figure 4, kidneys of OZR displayed greater accumulation of nitrotyrosine than those of the LZR; chronic Cr(pic)3 treatment was associated with attenuation of renal tissue nitrotyrosine levels thereby eliminating a significant differential in comparison to the LZR (Figure 4).

**Figure 5:**
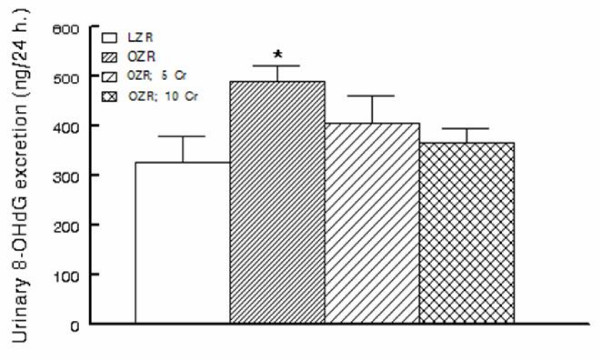
**Urinary excretion of 8-hydroxydeoxyguanosine (8-OHdG) in experimental groups as described in Figure 1**. Data are means ± SEM of 9-10 animals/group. * p < 0.05 compared to the LZR or OZR;10 Cr groups.

**Figure 6:**
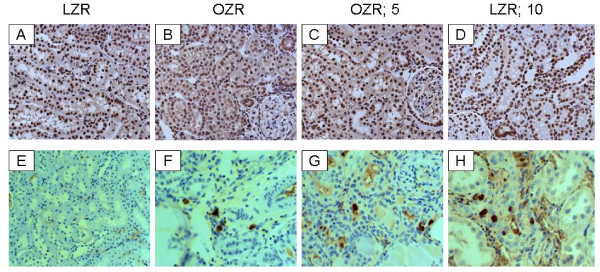
**Kidney 8-OHdG and CD 68 immunostaining**. Panels A-D show representative 8-OHdG-immunostained kidney sections from experimental groups while panels E-H show immunostaining for CD68 positive tissue histiocytes (200× except panel E which is shown at 100×).

In order to determine whether chronic Cr(pic)3 treatment increases whole body oxidative DNA damage, urinary excretion of 8-OHdG was measured in the experimental groups. As shown in Figure 5, the untreated OZR displayed greater urinary 8-OHdG excretion. The treatment was associated with a decline in urinary 8-OHdG excretion towards that of the LZR. Interestingly, however, immunostaining of renal tissue for 8-OHdG did not reveal differences in either the (nuclear) pattern or intensity of staining among experimental groups (Figure 6A-D); similar findings were noted for the pancreas (Figure 2M-P).

**Figure 7:**
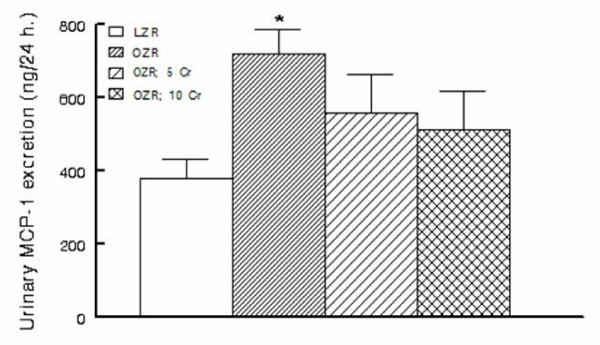
**Urinary excretion of MCP-1 in experimental groups as described in Figure 1**. Data are means ± SEM of 9-10 animals/group. * p < 0.05 compared to LZR group.

**Figure 8:**
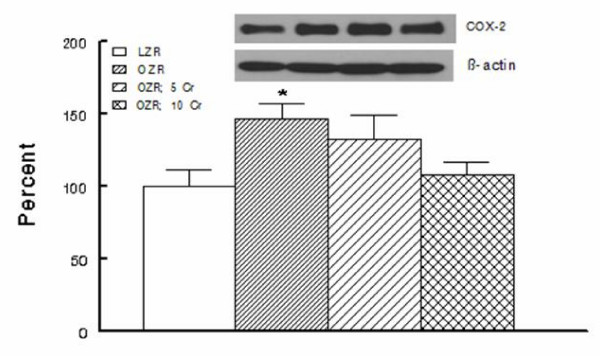
**Renal expression of cyclooxygensae-2 for experimental groups as described in Figure 1**. Data are normalized to β-actin and expressed as percent of the LZR group. Data are means ± SEM of 7-9 animals per group. Also shown is representative blot for each group and its β-actin control. * p < 0.05 compared to the LZR or OZR; 10 Cr groups.

As indices of inflammation, urinary excretion of MCP-1, immunostaining for tissue histiocytes (i.e., CD68 positive cells) and renal tissue level of COX-2 were determined. As shown in Figure 7, urinary excretion of MCP-1 was significantly higher in the OZR than LZR; Cr(pic)3 treatment was associated with attenuation of urinary MCP-1 excretion thereby eliminating the significant differential with respect to the LZR group. Examination of renal tissues for LZR group did not identify CD68 positive cells while those of the OZR, irrespective of Cr(pic)3 treatment showed generally similar foci of multiple CD68 positive cells (Figure 6E-H). Similarly, renal expression of COX-2 was increased in the OZR than LZR; the higher Cr(pic)3 diet was associated with reduction in COX-2 level towards that of the LZR group (Figure 8).

**Figure 9:**
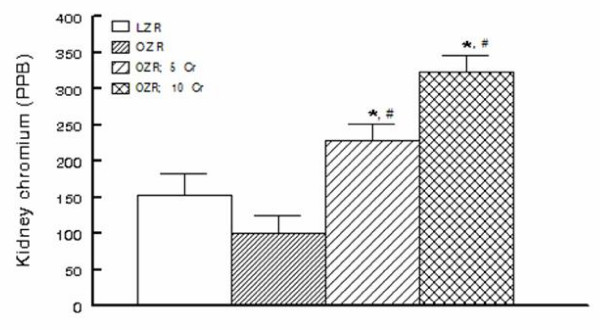
**Renal tissue chromium content in experimental groups as described in Figure 1**. PPB: Parts Per Billion. * p < 0.05 compared to either the LZR or untreated OZR groups. # p < 0.05 compared to other groups.

Chromium is known to accumulate in tissues including the kidney. Therefore, to determine whether the OZR kidney also accumulates chromium, renal tissue chromium content was measured by ICP-MS. As shown in Figure 9, renal tissue chromium content tended to be lower in the OZR than LZR, an aspect that may relate to the requirement of cellular uptake of chromium for normal insulin signaling [27]. As expected, dietary Cr(pic)3 treatment resulted in a dose-related accumulation of chromium in the OZR kidneys (Figure 9).

## Discussion

The present study shows that long-term Cr(pic)3 treatment does not influence abnormal glycemic status or indices of growth of OZR. Further, the treatment is not associated with exacerbated oxidative DNA damage. Rather, the treatment exerts mild-moderate beneficial effects on several markers of oxidative stress and inflammation in OZR.

The interest in chromium supplementation stems from earlier studies that suggested an essential role for trivalent chromium in carbohydrate and lipid metabolism [10,17,28]. These studies showed that rats consuming a diet lacking chromium developed an inability to efficiently dispose of blood glucose [17,10]. This defect was reversed by addition of chromium-enriched food or by supplementation with synthetic trivalent chromium. Subsequent observation that patients on total parenteral nutrition also develop a deficit in carbohydrate metabolism, which can be alleviated with trivalent chromium, established the essential role of trivalent chromium in human diet as well [17,10]. Although the exact molecular events subserving the effect of chromium on glucose metabolism remain to be established, a number of mechanisms have been proposed that collectively lead to amplification of insulin signaling (e.g., increased insulin receptor binding but inhibition of insulin receptor tyrosine phosphatase) [28,29]. In light of these reports, we had expected that long-term Cr(pic)3 treatment of OZR would improve insulin resistance. Surprisingly, however, the treatment did not beneficially influence several indices of glycemic control or the marked histopathological features of pancreatic islets in OZR. Nonetheless, the results are consistent with a recent report indicating that Cr(pic)3, at a dose of 1000 μg/day, does not improve insulin sensitivity or several other features of metabolic syndrome in obese adults [30]. Human studies have typically used chromium at a dose of 200-1000 μg/kg [29-31]. Assuming an average body weight of 70 kg, this corresponds to a range of about 3-14 μg/kg of chromium supplementation. Animal studies, on the other hand, have used dosages similar to (and even far higher than) the ones used in this study (about 190 to 410 μg/kg/day for OZR; 5 Cr and OZR; 10 Cr groups, respectively) [22,29,31]. Thus, it is likely that far higher doses of the formulation would be required to unmask a beneficial effect on glycemic control in the setting of marked insulin resistance as it occurs in OZR. Nonetheless, the results raise question about the efficacy of Cr(pic)3, alone, in doses that are commonly consumed by human subjects although the possibility that such doses may exert additive or synergistic effects with physical exercise and/or other interventional modalities remains to be explored. This notion is consistent with a report indicating that Cr(pic)3 supplementation increases insulin sensitivity and improves glycemic status of type 2 diabetic patients who are on sulfonylurea agents [32].

It is well-established that abnormal glycemic state of obesity and diabetes mellitus are associated with increased oxidative stress and subsequent oxidative DNA damage [3]; indeed, generation of 8-OHdG is considered as a surrogate marker of enhanced oxidative stress and associated oxidative DNA damage [33]. Thus, we conjectured that Cr(pic)3-induced improvement in glycemic control should reduce 8-OHdG production. On the other hand, if indeed Cr(pic)3 treatment increases oxidative DNA damage, then the treatment should further increase 8-OHdG generation in the setting of increased oxidative and nitrosative stress that are features of OZR [7]. Consistent with this notion, urinary excretion of 8-OHdG was significantly higher in the OZR than LZR, suggesting an increase in whole body oxidative stress. On the other hand, renal tissue nitrotyrosine level was increased in kidneys of OZR than LZR reflecting an increase in local oxidative/nitrosative stress. Interestingly, however, Cr(pic)3 treatment did not increase either urinary excretion of 8-OHdG or renal tissue nitrotyrosine content. Rather, the treatment was associated with mild reductions in both parameters that were sufficient enough to abrogate statistical significance compared to the LZR group. Interestingly, the reductions in 8-OHdG excretion and tissue nitrotyrosine level were not associated with any improvement in glycemic status of OZR. In this context, Chander and colleagues [7] have shown that treatment of OZR with ebselen (an antioxidant and peroxynitrite scavenger) reduces lipid peroxidative products and 3-nitrotyrosine-modified proteins without affecting blood glucose.

Since urinary excretion of 8-OHdG is a surrogate biomarker of whole body oxidative DNA damage, we also carried out immunohistochemical examination of the kidney for 8-OHdG. The rationale for this relates to the fact that trivalent chromium is known to accumulate in a number of organs although the kidney accumulates it to a greater extent [17-19]. Indeed, as shown in Figure 9, Cr(pic)3-enriched diets caused significant increases in kidney chromium content of the OZR. Thus, we conjectured that any adverse effect of Cr(pic)3 treatment on oxidative DNA damage should be more readily detected in the kidney. Interestingly, however, immunostaining of renal tissue did not reveal differential patterns or intensity among the experimental groups; similar findings were noted for the pancreas. These observations coupled with the demonstration that urinary excretion of 8-OHdG increases in conditions associated with oxidative stress suggest that the modified nucleoside is largely released rather than being accumulated in the tissue(s). Nonetheless, immunostaining findings and urinary excretion profile of 8-OHdG do not support the notion that Cr(pic)3 enhances oxidative DNA damage in OZR. As indicated earlier, a major concern regarding Cr(pic)3 relates to reports of increased risk of genotoxicity associated with its use [13-16]. Genotoxicity of trivalent chromium has been shown in acellular systems where direct interaction occurs with the genetic material [34,31,15]. However, trivalent chromium compounds, including Cr(pic)3, have produced conflicting results in ex vivo and in vivo studies likely due to multiple reasons including low cellular uptake of trivalent chromium and the demonstration that it does not avidly accumulate in DNA containing organelles such as the nucleus or the mitochondria [18,19]. The results of this study are in agreement with this notion and the more recent reports indicating lack of a significant DNA toxicity of Cr(pic)3 [20,35].

Consistent with other reports, the OZR displayed a number of renal abnormalities compared to their lean counterparts including greater albuminuria and histopathological changes indicative of extracellular matrix deposits, inflammatory infiltrates and tubulointerestitial injury. Nonetheless, renal tubular dilatations were noted for both LZR and OZR thereby suggesting lack of a correlation of this abnormality with metabolic derangements associated with obesity and (or) type 2 diabetes. It is noteworthy that there were numerous lipid droplets in renal tissue of OZR than LZR; ectopic lipid accumulation is believed to contribute importantly to pathophysiology of organ dysfunction in obesity/type 2 diabetes (i.e., lipotoxicity) [36]. More importantly, however, it is suggested that release of autocrine and paracrine factors from periorgan fat deposits contributes importantly to the pathogenesis of cardiac abnormalities in animals with dietary fat-induced obesity [37]. Indeed, OZR display marked deposits of fat around the kidney, effectively encasing the organ. Nonetheless, the contribution of autocrine and paracrine release of factors from adipose tissue surrounding the kidney, an encapsulated organ, to eventual manifestation of renal pathology in OZR remains to be established. Importantly, however, Cr(pic)3 treatment did not result in significant beneficial effects on renal function (e.g., albuminuria) or histopathological findings of OZR.

The lack of significant effects of Cr(pic)3 on renal function and structure are seemingly inconsistent with its mild-moderate ameliorating effects on indices of oxidative stress (e.g., tissue nitrotyrosine) and inflammation (e.g., urinary MCP-1 excretion and renal tissue COX-2 level). However, it is important to note that multiple mechanisms contribute to genesis of proteinuria and renal dysfunction associated with obesity and type 2 diabetes including metabolic, hemodynamic (both systemic and intrarenal), oxidative stress and a variety of inflammatory cytokines and chemokines [3]. Ultimately, the net effect of these changes results in loss of glomerular membrane permeability barrier and albuminuria. It is noteworthy that systemic hemodynamics and metabolic status of the 3 Cr(pic)3-treated OZR groups were generally similar thereby suggestive of similar contribution of these determinants to albuminuria. On the other hand, although the parameters that were measured in this study are known to contribute to genesis of proteinuria, it is likely that the mild Cr(pic)3-induced changes in their levels are not sufficient to beneficially influence albuminuria. Alternatively, Cr(pic)3 in doses that were used in this study may not be sufficient enough to alter the course and/or extent of proteinuria.

In conclusion, long-term Cr(pic)3 intake in doses that exceed those consumed by human subjects does not exert beneficial effects on glycemic control or adversely affect growth of OZR. In addition, despite renal accumulation of chromium, kidney function and structure were generally similar among untreated and treated OZR. Importantly, the treatment did not increase either urinary excretion of 8-OHdG or its immunohistochemical pattern and intensity in the kidney and the pancreas. Rather, Cr(pic)3 treatment of OZR was associated with attenuations in indices of oxidative stress and inflammation thereby diminishing the differentials with LZR.

## Competing interests

The authors declare that they have no competing interests.

## Authors' contributions

MSM is responsible for the design and the overall conduct of the study as well as data analysis and preparation of the manuscript. RA carried out histopathological examination of tissue samples. HW and JYL conducted the research (75% and 25% effort, respectively). AER provided instructions on slot blot analysis and AEM measured urinary MCP-1. The authors thank Noel Stanton of the Wisconsin State Laboratory of Hygiene for the analyses of kidney chromium content and Nutrition 21 for the generous gift of chromium picolinate. All authors read and approved the final manuscript.
